# Anti-Obesity Effects of the Flower of *Prunus persica* in High-Fat Diet-Induced Obese Mice

**DOI:** 10.3390/nu11092176

**Published:** 2019-09-11

**Authors:** Jungbin Song, Young-Sik Kim, Linae Kim, Hyo Jin Park, Donghun Lee, Hocheol Kim

**Affiliations:** 1 Department of Herbal Pharmacology, College of Korean Medicine, Kyung Hee University, 26 Kyungheedae-ro, Dongdaemun-gu, Seoul 02447, Korea; jbsong@khu.ac.kr (J.S.); yjbsik@gmail.com (Y.-S.K.); syusran@hanmail.net (L.K.); 2 Korea Institute of Science and Technology for Eastern Medicine (KISTEM), NeuMed Inc., 88 Imun-ro, Dongdaemun-gu, Seoul 02440, Korea; rnfyddyd@neumed.co.kr; 3 Department of Herbal Pharmacology, College of Korean Medicine, Gachon University, 1342 Seongnamdae-ro, Sujeong-gu, Seongnam-si, Gyeonggi-do 13120, Korea

**Keywords:** *Prunus persica*, peach blossom, high-fat diet, anti-obesity, glucose, liver, lipogenesis, β-oxidation

## Abstract

*Prunus persica* (L.) Batsch is a deciduous fruit tree cultivated worldwide. The flower of *P. persica* (PPF), commonly called the peach blossom, is currently consumed as a tea for weight loss in East Asia; however, its anti-obesity effects have yet to be demonstrated in vitro or in vivo. Since PPF is rich in phytochemicals with anti-obesity properties, we aimed to investigate the effects of PPF on obesity and its underlying mechanism using a diet-induced obesity model. Male C57BL/6 mice were fed either normal diet, high-fat diet (HFD), or HFD containing 0.2% or 0.6% PPF water extract for 8 weeks. PPF significantly reduced body weight, abdominal fat mass, serum glucose, alanine transaminase and aspartate aminotransferase levels, and liver and spleen weights compared to the HFD control group. Real-time quantitative polymerase chain reaction analysis revealed that PPF suppressed lipogenic gene expression, including stearoyl-CoA desaturase-1 and -2 and fatty acid synthase, and up-regulated the fatty acid β-oxidation gene, carnitine palmitoyltransferase-1, in the liver. Our results suggest that PPF exerts anti-obesity effects in obese mice and these beneficial effects might be mediated through improved hepatic lipid metabolism by reducing lipogenesis and increasing fatty acid oxidation.

## 1. Introduction

The global epidemic of overweight and obesity has become a major challenge for public health. Excessive adiposity leads to adverse metabolic consequences, including insulin resistance, chronic inflammation, and abnormal lipid metabolism, thereby increasing the risk of various diseases, such as cardiovascular diseases and diabetes [1]. Dietary and lifestyle interventions, i.e., restriction in calorie intake and increase in physical activity, are commonly recommended as an ideal strategy to counteract obesity development, but motivation and adherence are challenging [2]. Medications approved for overweight and obesity treatment include orlistat, lorcaserin, and liraglutide, and these drugs are intended only for patients with a body mass index (BMI) ≥ 30 or BMI ≥ 27 with comorbidities [3]. Because of a high incidence of side effects [3], anti-obesity drugs should be used only if the expected benefits outweigh the possible risks. As an alternative approach, many researchers have focused on the anti-obesity effects of natural ingredients that are consumed on a daily basis, because such ingredients are generally considered less toxic than synthetic drugs [4].

*Prunus persica* (L.) Batsch (Rosaceae family) is a deciduous fruit tree that originated in China and is now cultivated worldwide for its fruit, peach. The seeds, flowers, and leaves of *P. persica* have long been used as folk medicine in China and Korea [5,6,7]. In particular, the flower of *P. persica* (PPF), commonly called the peach blossom, has been used as a purgative, diuretic, and cosmetic [6,7]. Previous studies have revealed several pharmacological effects of PPF, including protection of the skin from ultraviolet radiation [8,9], inhibition of melanogenesis [10], stimulation of intestinal motility [6], and purgative action [11]. PPF is currently consumed as a tea for weight loss in China and Korea; however, its anti-obesity effects have yet to be demonstrated in vitro or in vivo.

PPF is rich in flavonoids and phenolic phytochemicals, the major compounds being chlorogenic acid, kaempferol and its derivatives (including multiflorin A and B, astragalin, afzelin, and trifolin), and quercetin derivatives (such as quercitrin, isoquercitrin, rutin, hyperoside, hyperin, and multinoside A and B) [8,9,11,12,13,14]. Many of these compounds have been reported to possess anti-obesity properties in vitro or in vivo. Chlorogenic acid reduced adiposity and improved lipid metabolism in diet-induced obese mice [15]. Astragalin, isoquercitrin, and hyperoside increased lipolysis in the adipose tissue of mice [16]. Kaempferol, quercitrin, and isoquercitrin have been shown to inhibit adipogenesis in 3T3-L1 adipocytes [17,18,19]. Rutin inhibited adiposity, increased energy expenditure, and improved glucose homeostasis in obese mice [20]. In addition, multiflorin A and hyperin have shown anti-hyperglycemic activities in rodents [21,22]. These previous studies propose that PPF may be effective in reducing obesity and related metabolic disorders.

The present study aimed to determine whether PPF exerts anti-obesity effects in a diet-induced obesity model. Male C57BL/6 mice were fed a high-fat diet (HFD) with or without PPF water extract for 8 weeks, and the body weight, fat mass, organ weights, and serum biochemical profiles were measured. We also explored the molecular mechanism underlying the effects of PPF on lipid metabolism in the liver of HFD-fed mice.

## 2. Materials and Methods 

### 2.1. Sample Preparation

The dried flowers of *P. persica* were purchased from Xian Chinese herb market (Shaanxi province, China) and authenticated by Professor Hocheol Kim. The flower was first extracted with 20 times (*v*/*w*) the amount of distilled water at 100 °C for 2 h under reflux and secondly extracted with 15 times (*v*/*w*) the amount of distilled water in the same manner. The extract was filtered, concentrated under reduced pressure, and lyophilized to yield a power (extraction yield 28.5%). A voucher specimen of the raw material was deposited in the Herbarium of Kyung Hee University, College of Korean Medicine.

### 2.2. High-Performance Liquid Chromatography (HPLC) Analysis

HPLC analysis was performed on an Agilent 1220 Infinity LC System (Agilent technologies, Santa Clara, CA, USA) for quantifying prunin, the marker compound of PPF extract [23]. A reverse-phase Agilent Eclipse XDB-C_18_ column (4.6 mm × 250 mm, 5 μm) was used and maintained at 40 °C. The mobile phase consisting of 0.1% phosphoric acid (A) and acetonitrile (B) was used at a flow rate of 0.5 mL/min. The gradient elution conditions for detecting prunin were 15–15% B (0–5 min), 15–20% B (5–15 min), 20–20% B (15–38 min), 20–15% (38–39 min), and 15–15% (39–40 min). The injection volume was 10 μL, and the detection was monitored at 288 nm.

### 2.3. Animals and Diets

Male C57BL/6 mice (3 weeks old) obtained from Orient Bio Inc. (Seongnam, Republic of Korea) were housed in standard laboratory conditions (23 ± 1 °C, 55% ± 5% humidity, and 12 h light/dark cycle). Male mice were chosen since they have a higher susceptibility to obesity than female mice [24]. After a week’s acclimation period, the mice were randomly divided into four groups with twelve mice in each group: normal diet (ND), HFD control, HFD + 0.2% PPF, and HFD + 0.6% PPF groups. The ND group received standard chow for 8 weeks, while the HFD control group received an HFD with 60% kcal fat (Research Diets Inc., New Brunswick, NJ, USA, Cat. No. D12492). The HFD + 0.2% PPF and HFD + 0.6% PPF groups received a HFD containing 0.2% and 0.6% PPF extract, which were equivalent to approximately 200 and 600 mg/kg/day, respectively. All mice were given free access to food and distilled water. Their food intake and body weight were measured twice a week. All mice were checked once daily for clinical signs during the experimental period. After 8 weeks, all mice were fasted for 12 h before sacrifice and blood samples were collected from the inferior vena cava under 2% isoflurane anesthesia. Liver, spleen, and abdominal adipose tissues (epididymal, perirenal, and mesenteric) were quickly removed and weighed. The blood samples were kept at room temperature for 30 min, and then centrifuged at 3000 rpm for 10 min at 4 °C to obtain the serum. The liver tissue was stored at −80 °C for further analysis. The animal experimental protocols were approved by the International Animal Care and Use Committee of Korea, Institute of Science and Technology for Eastern Medicine (Approval No. KISTEM-IACUC-2016-002). 

### 2.4. Serum Biochemical Analysis

Serum levels of glucose, triglyceride, total cholesterol, alanine aminotransferase (ALT), and aspartate aminotransferase (AST) were analyzed using VetTest 8008 (IDEXX Lab Inc., Westbrook, ME, USA) according to the manufacturer’s instruction.

### 2.5. Real-Time Quantitative PCR Analysis

Total RNAs were isolated from the liver using QIAzol lysis reagent (Qiazen, Venlo, Netherlands, Cat no. 79306), and then reverse-transcribed into cDNA using the high capacity cDNA reverse transcription kit (Applied Biosystems, Waltham, MA, USA, Cat. No. 4368814) according to the manufacturer’s instruction. The mRNA expression was quantified by the StepOnePlus™ Real-Time PCR System (Applied Biosystems Inc., MA, USA) using the SYBR green PCR Master Mix (Applied Biosystems Inc., MA, USA, Cat. No. 4367659). The primer sequences were as follows: 5′-CTGACCTACTACTTCAAGGGCAGT-3′ and 5′-GGGAGTCTGTATGAATACCTCTGC-3′ for stearoyl-CoA desaturase (SCD)-1; 5′-GTAGTGGTACAGTGCTGCTGAAAG-3′ and 5′-CTCTTCCTGAAGTGAGGTCCAT-3′ for stearoyl-CoA desaturase (SCD)-2; 5′-GTACAGGCTGAAGGAGGACACT-3′ and 5′-TGAGATGTGGATACCACCAGAG-3′ for fatty acid synthase (FAS); 5′-ACAGTCCAGCCTTTGAGGATAG-3′ and 5′-GACACAGAAAGGCCAGTACACA-3′ for sterol regulatory element-binding protein (SREBP)-1c; 5′-CAGTGGGGAGAGAGGACAGA -3′ and 5′-AGTTCGGGAACAAGACGTTG-3′ for peroxisome proliferator-activated receptor (PPAR) α; 5′-CCAGGCTACAGTGGGACATT-3′ and 5′-GAACTTGCCCATGTCCTTGT-3′ for carnitine palmitoyltransferase (CPT)-1; and 5′-ACAATGAATACGGCTACAGCAACAG-3′ and 5′-GGTGGTCCAGGGTTTCTTACTCC-3′ for glyceraldehyde-3-phosphate dehydrogenase. The relative gene expression was calculated with the 2^−△△Ct^ method.

### 2.6. Statistical Analysis

All data are presented as mean ± standard deviation. The differences between more than two groups were compared by one-way analysis of variance (ANOVA) followed by Tukey’s multiple comparison test. The difference between the two groups was compared by independent *t*-test. Statistical analysis was performed using Prism 5 software (GraphPad Software, Inc., San Diego, CA, USA). A probability value of *p* < 0.05 was considered statistically significant. 

## 3. Results

### 3.1. HPLC Analysis

**Figure 1:**
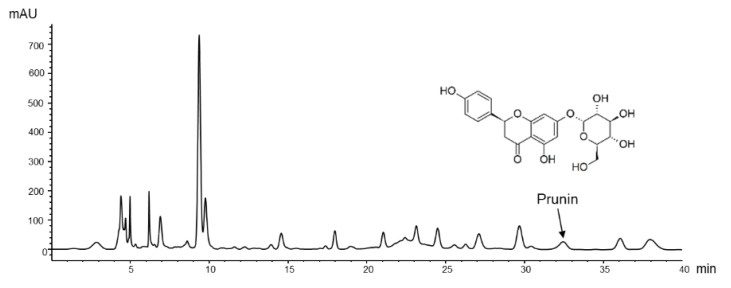
High-performance liquid chromatography (HPLC) chromatogram of *P. persica* flower extract. The monitoring wavelength for prunin was set at 288 nm.

The HPLC chromatogram of PPF extract is shown in Figure 1. Prunin was detected at a retention time of 32.5 min and quantified as 2.59 mg/g of dried extract.

### 3.2. Effects of P. persica Flower on Body Weight and Food Intake

**Figure 2:**
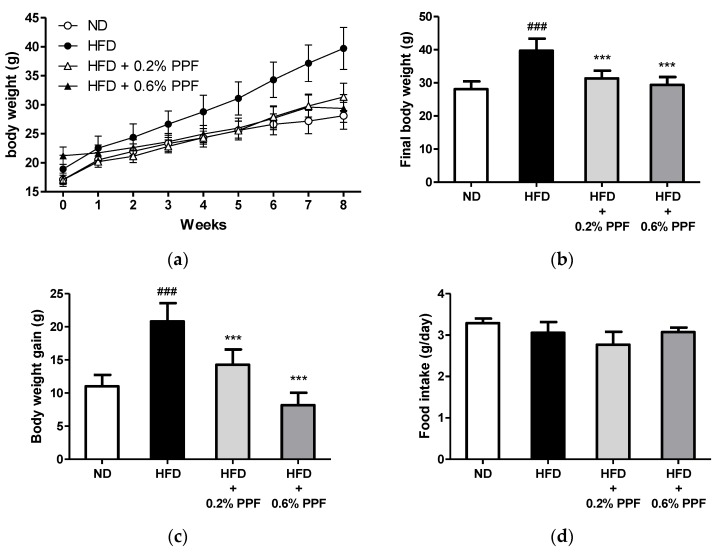
Effects of *P. persica* flower on body weight and food intake in high-fat diet-fed C57BL/6 mice. (**a**) Changes in body weight of mice fed either normal diet (ND), high-fat diet (HFD), or HFD containing 0.2% or 0.6% *P. persica* flower (PPF) extract for eight weeks; (**b**) final body weight; (**c**) body weight gain during 8 weeks of supplementation; (**d**) mean daily food intake. Values are expressed as mean ± SD (*n* = 12/group). ### *p <* 0.001 vs. ND group. *** *p* < 0.001 vs. HFD control group.

The administration of an HFD for 8 weeks significantly increased the body weight and body weight gain compared to the ND group (both *p <* 0.001, Figure 2a–c). In contrast, mice fed an HFD containing 0.2% and 0.6% PPF extract had markedly decreased body weight and body weight gain at 8 weeks compared with those in the HFD control group (all *p <* 0.001, Figure 2b,c). There were no significant differences in food intake between the groups (Figure 2d). In addition, mice treated with PPF showed no abnormal clinical signs during the study period.

### 3.3. Effects of P. persica Flower on Abdominal Fat Weights

**Figure 3:**
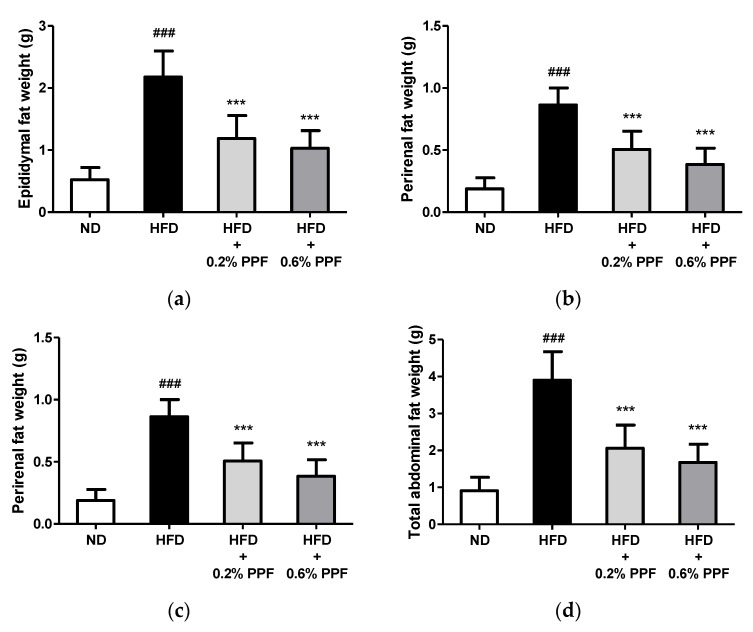
Effects of *P. persica* flower on epididymal (**a**), perirenal (**b**), mesenteric (**c**), and total abdominal (**d**) fat weights in high-fat diet-fed C57BL/6 mice. Mice were fed either normal diet (ND), high-fat diet (HFD), or HFD containing 0.2% or 0.6% *P. persica* flower (PPF) extract for eight weeks. Total abdominal fat (**d**) was calculated as the sum of the three abdominal depots (**a**–**c**). Values are expressed as mean ± SD (*n* = 12/group). ### *p <* 0.001 vs. ND group. *** *p* < 0.001 vs. HFD control group.

After eight weeks of feeding, abdominal fat weights in the HFD control group were increased by approximately four times the levels found in the ND group, confirming that HFD resulted in obesity in mice (all *p <* 0.001, Figure 3a–d). The supplementation with 0.2% and 0.6% PPF extract significantly reduced the total and individual (epididymal, perirenal, and mesenteric) fat pad weights compared to the HFD control group (all *p <* 0.001).

### 3.4. Effects of P. persica Flower on Serum Biochemical Profiles

HFD feeding significantly increased serum glucose, total cholesterol, ALT, and AST levels compared to those in the ND group (all *p <* 0.001, Table 1), while triglyceride levels were not altered. The serum glucose levels were significantly reduced in the HFD + 0.2% PPF and HFD + 0.6% PPF groups in a dose-dependent manner compared to those of the HFD group (both *p <* 0.001). There were no significant differences between the HFD and HFD + PPF groups in the total cholesterol and triglyceride levels. In contrast, ALT and AST levels were significantly reduced by supplementation with 0.2% PPF extract (both *p <* 0.01) as well as 0.6% PPF extract (*p <* 0.001 and *p <* 0.05, respectively). 

### 3.5. Effects of P. persica Flower on Liver and Spleen Weights

**Figure 4:**
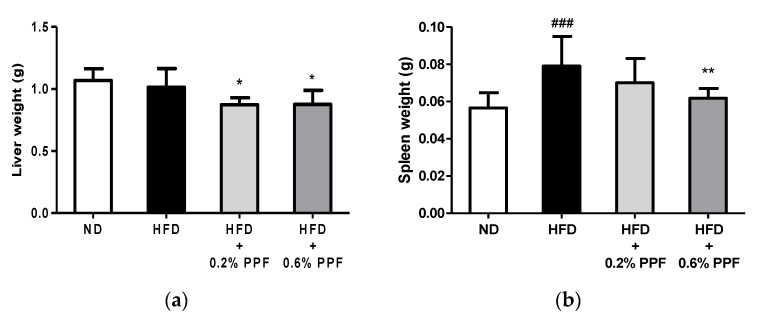
Effects of *P. persica* flower on liver (**a**) and spleen (**b**) weights in high-fat diet-fed C57BL/6 mice. Mice were fed either normal diet (ND), high-fat diet (HFD), or HFD containing 0.2% or 0.6% *P. persica* flower (PPF) extract for eight weeks. Values are expressed as mean ± SD (*n* = 12/group). ### *p <* 0.001 vs. ND group. * *p* < 0.05 and ** *p* < 0.01 vs. HFD control group.

There was no difference in the liver weight between the ND and HFD groups at eight weeks (Figure 4a). Nonetheless, HFD mice supplemented with 0.2% and 0.6% PPF extract showed a significant decrease in the liver weight compared to the HFD control mice (both *p <* 0.05). The administration of HFD increased the spleen weight compared to the ND group (*p <* 0.001, Figure 4b), while the supplementation with 0.6% PPF extract significantly reduced the spleen weight compared to the HFD group (*p <* 0.01).

### 3.6. Effects of P. persica Flower on Lipogenesis-Related Gene Expression in the Liver

**Figure 5:**
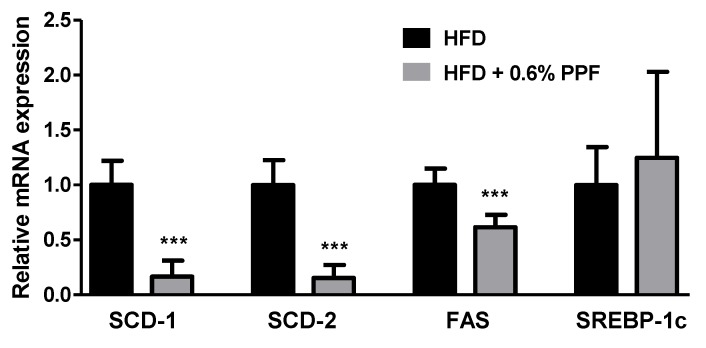
Effects of *P. persica* flower on hepatic lipogenic gene expression in high-fat diet-fed C57BL/6 mice. The mRNA expression levels of stearoyl-CoA desaturase (SCD)-1 and -2, fatty acid synthase (FAS), and sterol regulatory element-binding protein 1c (SREBP-1c) were measured in the liver. Mice were fed either high-fat diet (HFD) or HFD containing 0.6% *P. persica* flower (PPF) extract for eight weeks. Values are expressed as mean ± SD (*n* = 12/group). The y-axis is relative expression level normalized against the HFD control group. *** *p* < 0.001 vs. HFD control group.

To investigate the mechanisms underlying the effects of PPF, we compared the expression of genes involved in hepatic lipid metabolism between the HFD and HFD + PPF groups. Since high-dose (0.6%) PPF was more effective than low-dose (0.2%) at reducing adiposity, the HFD + 0.6% PPF group was selected for further comparison. The mRNA levels of hepatic lipogenic enzymes, including SCD-1, SCD-2, and FAS were significantly decreased in the HFD + 0.6% PPF group compared to the HFD control group (all *p <* 0.001, Figure 5), while there was no difference in the mRNA level of SREBP-1c between the two groups.

### 3.7. Effects of P. persica Flower on Fatty Acid Oxidation-Related Gene Expression in the Liver

**Figure 6:**
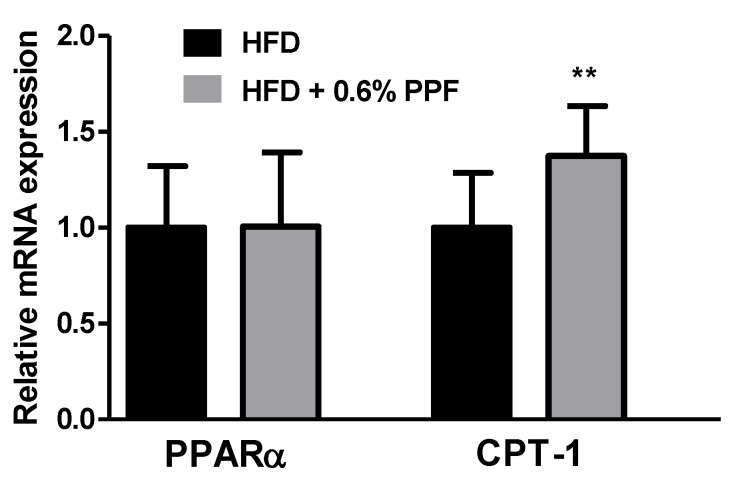
Effects of *P. persica* flower on β-oxidation-related gene expression. The mRNA expression levels of peroxisome proliferator-activated receptor α (**a**) and carnitine palmitoyltransferase-1 (**b**) were measured in the liver. Mice were fed either high-fat diet (HFD) or HFD containing 0.6% *P. persica* flower (PPF) extract for eight weeks. Values are expressed as mean ± SEM. The y-axis is relative expression level normalized against the HFD control group. ** *p* < 0.01 vs. HFD control group. *n* = 12/group.

To investigate the role of PPF on β-oxidation process, we measured the gene expression of PPARα and CPT-1. There was no difference in the mRNA level of PPARα between the two groups (Figure 6), while CPT-1 mRNA level was significantly increased in the HFD + 0.6% PPF group compared to the HFD control group (*p <* 0.01). 

## 4. Discussion

This study is the first, to our knowledge, to demonstrate that PPF was effective at reducing adiposity in HFD-fed obese mice. In addition, PPF attenuated HFD-induced pathological changes in the blood, liver, and spleen, and regulated lipid metabolism gene expression in the liver.

PPF significantly suppressed HFD-induced body weight gain and abdominal fat accumulation without change in food consumption. White adipose tissue is the major site of lipid storage, and its main depots in rodents include epididymal, perirenal, and mesenteric pads [25], which were all reduced by PPF. Excessive visceral fat causes the release of free fatty acids into the circulation, insulin resistance, and secretion of inflammatory adipokines, thereby raising the risk of metabolic diseases such as diabetes and non-alcoholic fatty liver disease [26]. It has been speculated that mesenteric fat in rodents most closely resembles human visceral fat owing to its high vascularity and access to the portal drainage system [27,28]. Notably, high-dose (0.6%) PPF reduced the mesenteric fat weight to levels comparable to those in the ND group. Our results suggest that PPF exhibits potent anti-obesity effects without affecting the appetite.

It is well known that obesity is accompanied by abnormal glucose and lipid metabolism. When C57BL/6 mice are fed with HFD, hyperglycemia and hypercholesterolemia are initially evident (within four weeks) while triglyceride levels are not elevated until much later (35 weeks) [29,30,31], which was consistent with our findings. Herein, the elevated glucose levels were markedly reduced to normal levels after PPF treatment, with levels in the 0.6% PPF group being lower than those in the ND group, indicating its hypoglycemic activity. Previous studies have reported that multiflorin A, one of the major component of PPF, reduced postprandial blood glucose level in glucose-loaded mice via inhibition of glucose absorption in the small intestine [11,21], and hyperin, another PPF component, exhibited anti-hyperglycemic activity in streptozotocin-induced diabetic rats [22]. Our results, along with previous evidence, indicate that PPF ameliorates obesity-induced hyperglycemia.

PPF significantly lowered ALT and AST level elevations and liver and spleen weights in HFD-fed mice. Long-term HFD consumption leads to hepatic lipid accumulation and development of fatty liver and non-alcoholic fatty liver disease, which is evidenced by increased ALT and AST levels [32]. The elevated liver enzyme levels are regarded as a surrogate marker of hepatic lipid accumulation [33]. HFD also induces spleen enlargement via sinusoidal dilatation and extracellular deposits, such as lipid and hemosiderin [34,35]. This enlarged spleen, as the main immune organ, produces higher levels of inflammatory cytokines and shows increased T cell proliferation, contributing to chronic systemic inflammation [36]. Our results suggest that PPF might counteract obesity-induced fatty liver and splenomegaly.

The liver is an important organ involved in lipid metabolism. In obese individuals, the hepatic triglyceride synthesis rate exceeds the rate of hepatic triglyceride catabolism, which leads to fat accumulation in the liver, insulin resistance, and exacerbation of pre-existing obesity [37]. PPF significantly decreased the hepatic mRNA expression of lipogenic genes, SCD-1 and -2 as well as FAS, compared with those in the HFD control mice. Hepatic triglycerides are synthesized by elongation and subsequent desaturation of fatty acids provided either by the uptake of plasma free fatty acid or produced by de novo lipogenesis [37]. SCD and FAS are the key enzymes regulating these processes, and their gene expressions are well known to increase markedly in the liver of mice fed HFD [38,39]. SCD, the rate-limiting enzyme that catalyzes the crucial committing step in the biosynthesis of monounsaturated fatty acids from saturated fatty acids, has been shown to be an important regulatory factor in hepatic lipogenesis and body fat regulation [40,41]. Targeted disruption in the SCD-1 and SCD-2 has been reported to protect mice against HFD-induced adiposity [42,43,44]. Particularly, SCD-1, as the main isoform expressed in the liver, has been proposed as a therapeutic target for obesity [42]. FAS is regarded as an important determinant of the maximal hepatic capacity for fatty acid synthesis by de novo lipogenesis because it catalyzes the final step of fatty acid biosynthesis [45]. A previous study reported that when fed HFD diet, only a small portion (1%) of hepatic triglycerides are derived from de novo lipogenesis [46]. Thus, it is presumed that SCD inhibition rather than FAS inhibition contributed more to the anti-obesity effects of PPF. Overall, our findings suggest that the anti-obesity effects of PPF can be mediated via inhibition of hepatic lipogenesis

PPARα is a key transcription factor that regulates the gene expression involved in peroxisomal and mitochondrial β-oxidation in the liver [47]. PPF did not alter the PPARα mRNA level, suggesting that activation of PPARα may not be necessary for the anti-obesity effects of PPF. Interestingly, PPF significantly increased the mRNA expression of CPT-1, a known target gene of PPARα. CPT-1 is responsible for the transport of activated fatty acids into the mitochondria for β-oxidation [48]. Recent evidence has demonstrated that hepatic gene expression of fatty acid oxidation enzymes, such as CPT-1, is induced by SCD-1 deficiency without PPARα activation [43,49]. Considering that PPF reduced SCD-1 expression by approximately 80% in this study, the increase in CPT-1 expression by PPF can be attributed to the change in SCD-1 expression. Our results suggest that increased hepatic fatty acid oxidation could also contribute to the anti-obesity effects of PPF.

The strength of our study is that we provided scientific support to the current use of PPF for weight loss. A caveat is that we only measured mRNA levels, which do not necessarily correlate with protein levels. Future studies are proposed to develop PPF as an anti-obesity agent. Herein, we focused mainly on the adipose tissue and liver. The role of PPF in other metabolic organs involved in obesity could be investigated. In addition, we confirmed the anti-obesity effects of PPF in only male mice, which are more prone to obesity than female mice [24]. A future study is expected to investigate the anti-obesity effects in females. Third, the effects of PPF on obesity-induced inflammation could be examined since its major components, chlorogenic acid and derivatives of kaempferol and quercetin, are well known to attenuate inflammatory responses [50,51,52]. Furthermore, although PPF has long been consumed as a tea, toxicity and pharmacokinetic studies are warranted to exclude safety concerns and to better understand kinetic behaviors.

## 5. Conclusions

Our results demonstrate that PPF has anti-obesity effects accompanying the decrease in obesity-induced hyperglycemia and liver and spleen damage. These beneficial effects might be mediated by reduced lipogenesis and increased fatty acid oxidation in the liver. Our findings provide evidence to support the potential health benefits of PPF in managing obesity and obesity-associated metabolic disorders.

## Figures and Tables

**Table 1:** Serum biochemical profiles after eight weeks of supplementation with *P. persica* flower extract.

Groups	Glucose (mg/dL)	TC (mg/dL)	Triglyceride (mg/dL)	ALT (U/L)	AST (U/L)
ND	179.3 ± 6.1 ^1^	77.5 ± 6.7	68.5 ± 13.0	38.6 ± 13.6	35.6 ± 8.9
HFD	239.6 ± 27.1 ^###^	106.8 ± 18.2 ^###^	71.8 ± 16.4	61.8 ± 23.1 ^###^	61.4 ± 13.8 ^###^
HFD + 0.2% PPF	178.9 ± 38.8 ***	105.9 ± 13.8	87.0 ± 29.7	38.0 ± 8.9 **	44.8 ± 8.1 **
HFD + 0.6% PPF	136.6 ± 31.9 ***	101.8 ± 17.1	84.3 ± 26.7	36.2 ± 10.3 ***	49.0 ± 14.0 *

^1^ Values are expressed as mean ± SD (*n* = 12/group). ### *p <* 0.001 vs. ND group. * *p* < 0.05, ** *p* < 0.01, and *** *p* < 0.001 vs. HFD control group. ALT, alanine aminotransferase; AST, aspartate aminotransferase; HFD, high-fat diet; ND, normal diet; PPF, *Prunus persica* flower; TC, total cholesterol.
